# Two Distinct E3 Ubiquitin Ligases Have Complementary Functions in the Regulation of Delta and Serrate Signaling in *Drosophila*

**DOI:** 10.1371/journal.pbio.0030096

**Published:** 2005-03-15

**Authors:** Roland Le Borgne, Sylvie Remaud, Sophie Hamel, François Schweisguth

**Affiliations:** **1**Ecole Normale Supérieure, CNRS UMR 8542ParisFrance; Cambridge UniversityUnited Kingdom

## Abstract

Signaling by the Notch ligands Delta (Dl) and Serrate (Ser) regulates a wide variety of essential cell-fate decisions during animal development. Two distinct E3 ubiquitin ligases, Neuralized (Neur) and Mind bomb (Mib), have been shown to regulate Dl signaling in Drosophila melanogaster and *Danio rerio,* respectively. While the *neur* and *mib* genes are evolutionarily conserved, their respective roles in the context of a single organism have not yet been examined. We show here that the *Drosophila mind bomb (D-mib)* gene regulates a subset of Notch signaling events, including wing margin specification, leg segmentation, and vein determination, that are distinct from those events requiring *neur* activity. *D-mib* also modulates lateral inhibition, a *neur*- and *Dl*-dependent signaling event, suggesting that D-mib regulates Dl signaling. During wing development, expression of *D-mib* in dorsal cells appears to be necessary and sufficient for wing margin specification, indicating that D-mib also regulates Ser signaling. Moreover, the activity of the *D-mib* gene is required for the endocytosis of Ser in wing imaginal disc cells. Finally, ectopic expression of *neur* in *D-mib* mutant larvae rescues the wing *D-mib* phenotype, indicating that Neur can compensate for the lack of *D-mib* activity. We conclude that D-mib and Neur are two structurally distinct proteins that have similar molecular activities but distinct developmental functions in *Drosophila*.

## Abstract 2

The Notch pathway is an important mechanism for communication between cells. In this paper, the roles of two related proteins in the Notch pathway are unravelled

## Introduction

Cell-to-cell signaling mediated by receptors of the Notch (N) family has been implicated in various developmental decisions in organisms ranging from nematodes to mammals [1]. N is well-known for its role in lateral inhibition, a key patterning process that organizes the regular spacing of distinct cell types within groups of equipotent cells. Additionally, N mediates inductive signaling between cells with distinct identities. In both signaling events, N signals via a conserved mechanism that involves the cleavage and release from the membrane of the N intracellular domain that acts as a transcriptional co-activator for DNA-binding proteins of the CBF1/Suppressor of Hairless/Lag-2 (CSL) family [2].

Two transmembrane ligands of N are known in *Drosophila,* Delta (Dl) and Serrate (Ser) [3]. Dl and Ser have distinct functions. For instance, *Dl* (but not *Ser*) is essential for lateral inhibition during early neurogenesis in the embryo [4]. Conversely, *Ser* (but not *Dl*) is specifically required for segmental patterning [5]. Some developmental decisions, however, require the activity of both genes: *Dl* and *Ser* are both required for the specification of wing margin cells during imaginal development [6,7,8,9,10]. These different requirements for *Dl* and *Ser* appear to primarily result from their non-overlapping expression patterns rather than from distinct signaling properties. Consistent with this interpretation, *Dl* and *Ser* have been proposed to act redundantly in the sensory bristle lineage where they are co-expressed ([11]; note, however, that results from another study have indicated a non-redundant function for *Dl* in the bristle lineage [12]). Furthermore, Dl and Ser appear to be partially interchangeable because the forced expression of Ser can partially rescue the *Dl* neurogenic phenotype [13]. Additionally, the ectopic expression of Dl can partially rescue the *Ser* wing phenotype [14]. The notion that Dl and Ser have similar signaling properties has, however, recently been challenged by the observation that human homologs of Dl and Ser have distinct instructive signaling activity [15].

Endocytosis has recently emerged as a key mechanism regulating the signaling activity of Dl. First, clonal analysis in *Drosophila* has suggested that dynamin-dependent endocytosis is required not only in signal-receiving cells but also in signal-sending cells to promote N activation [16]. Second, mutant Dl proteins that are endocytosis defective exhibit reduced signaling activity [17]. Third, two distinct E3 ubiquitin ligases, Neuralized (Neur) and Mind bomb (Mib), have recently been shown to regulate Dl endocytosis and N activation in *Drosophila* and *Danio rerio,* respectively [18,19,20,21,22,23,24,25]. Ubiquitin is a 76-amino-acid polypeptide that is covalently linked to substrates in a multi-step process that involves a ubiquitin-activating enzyme (E1), a ubiquitin-conjugating enzyme (E2), and a ubiquitin–protein ligase (E3). E3s recognize specific substrates and catalyze the transfer of ubiquitin to the protein substrate. Ubiquitin was first identified as a tag for proteins destined for degradation. More recently, ubiquitin has also been shown to serve as a signal for endocytosis [26,27]. Mib in D. rerio and Neur in *Drosophila* and *Xenopus* have been shown to associate with Dl, regulate Dl ubiquitination, and promote its endocytosis [18,19,20,22,25,28]. Moreover, genetic and transplantation studies have indicated that both Neur and Mib act in a non-autonomous manner [18,21,22,23,25,29], indicating that endocytosis of Dl is associated with increased Dl signaling activity. Finally, epsin, a regulator of endocytosis that contains a ubiquitin-interacting motif and that is known in *Drosophila* as Liquid facet, is essential for Dl signaling [30,31]. In one study, Liquid facet was proposed to target Dl to an endocytic recycling compartment, suggesting that recycling of Dl may be required for signaling. Accordingly, signaling would not be linked directly to endocytosis, but endocytosis would be prerequisite for signaling [30]. How endocytosis of Dl leads to the activation of N remains to be elucidated. Also, whether the signaling activity of Ser is similarly regulated by endocytosis is not known.

Neur and Mib proteins completely differ in primary structure. *Drosophila* Neur is a 754-amino-acid protein that contains two conserved Neur homology repeats of unknown function and one C-terminal catalytic really interesting new gene (RING) domain. D. rerio Mib (also known as DIP-1 in the mouse [32]) is a 1,030-amino-acid protein with one ZZ zinc finger domain surrounded by two Mib/HERC2 domains, two Mib repeats, eight ankyrin repeats, two atypical RING domains, and one C-terminal catalytic RING domain. Both genes have been conserved from flies to mammals [18,19,33,34]. While genetic analysis has revealed that *neur* in *Drosophila* and *mib* in D. rerio are strictly required for N signaling, knockout studies of mouse *Neur1* has indicated that NEUR1 is not strictly required for N signaling [33,34]. One possible explanation is functional redundancy with the mouse *Neur2* gene. Conversely, the function of *Drosophila mib (D-mib),* the homolog of *D. rerio mib* gene, is not known.

To establish the respective roles of these two distinct E3 ligases in the context of a single model organism, we have studied the function of the *Drosophila D-mib* gene. We report here that D-mib, like D. rerio Mib, appears to regulate Dl signaling during leg segmentation, wing vein formation, and lateral inhibition in the adult notum. We further show that *D-mib* is specifically required for Ser endocytosis and signaling during wing development, indicating for the first time, to our knowledge, that endocytosis regulates Ser signaling. Interestingly, the *D-mib* activity was found necessary for a subset of N signaling events that are distinct from those requiring the activity of the *neur* gene. Nevertheless, the ectopic expression of Neur compensates for the loss of *D-mib* activity in the wing, indicating that Neur and D-mib have overlapping functions. We conclude that D-mib and Neur are two structurally distinct proteins with similar molecular activities but distinct and complementary functions in *Drosophila*.

## Results

### Isolation of *D-mib* Mutations

The closest *Drosophila* homolog of the vertebrate *mib* gene is the predicted gene CG5841, *D-mib* [18]. The *D-mib* mutations identified are shown in Figure 1. A P-element inserted into the 5′ untranslated region of the *D-mib* gene was recently isolated (http://flypush.imgen.bcm.tmc.edu/pscreen/) (Figure 1A). Insertion of this P-element confers late pupal lethality. Lethality was reverted by precise excision of the P-element, suggesting that insertion of this P-element is a *D-mib* mutation, referred to as *D-mib^1^*. A 13.6-kb deletion that removes the entire *D-mib* coding region was selected by imprecise excision of this P-element. This deletion represents a null allele of *D-mib* and was named *D-mib^2^*. This deletion also deletes the 3′ flanking *RpS31* gene (Figure 1A). The *D-mib^1^* and *D-mib^2^*mutant alleles did not complement the *l(3)72Cda^J12^* and *l(3)72Cda^I5^* lethal mutations that have been mapped to the same cytological interval as the *D-mib* gene [35]. This indicates that these two lethal mutations are *D-mib* mutant alleles, and they were therefore renamed *D-mib^3^* and *D-mib^4^,* respectively. The *D-mib^1^* and *D-mib^3^* mutations behave as genetic null alleles (see Materials and Methods). In contrast, *D-mib^4^* is a partial loss-of-function allele because flies *trans*-heterozygous for *D-mib^4^* and any other *D-mib* null alleles are viable.

**Figure 1:**
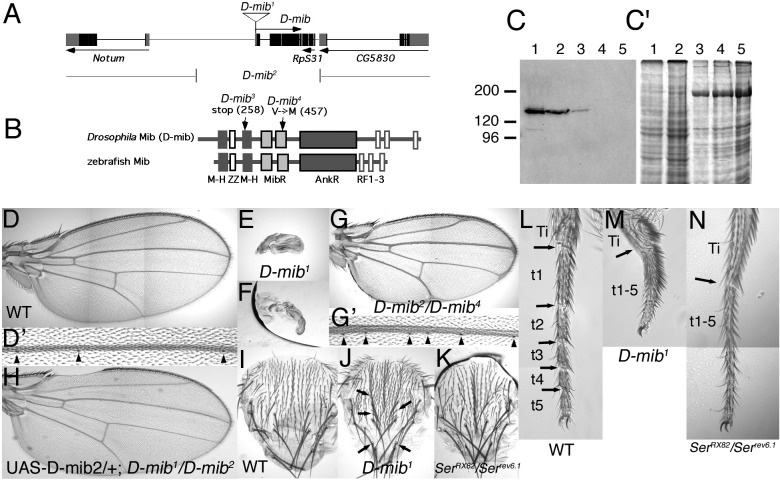
Molecular and Genetic Characterization of *D-mib* Mutations (A) Molecular map of the *D-mib* locus showing the position of the P-element inserted into the 5′ untranslated region (allele *D-mib^1^*) and the 13.6 kb deletion that removes the *D-mib* and the *RpS31* genes (allele *D-mib^2^*). Transcribed regions are indicated with arrows, and exons are indicated with boxes. Open reading frames are shown in black. (B) Domain composition of D-mib and D. rerio Mib. Both proteins show identical domain organization. D-mib has an N-terminal ZZ zinc finger flanked on either side by a Mib/HERC2 (M-H) domain, followed by two Mib repeats, six ankyrin repeats, two atypical RING domains, and a C-terminal protypical RING that has been associated with catalytic E3 ubiquitin ligase activity. The *D-mib^3^* mutant allele is predicted to produce a truncated protein devoid of E3 ubiquitin ligase activity whereas the *D-mib^4^* protein carries a mutation at a conserved position in the second Mib repeat. (C and C′) Western blot analysis of D-mib (C). The endogenous D-mib protein (predicted size: 130 kDa) was detected in S2 cells (lane 2) and in imaginal discs from wild-type larvae (lane 3) but was not detectable in homozygous *D-mib^1^* (lane 4) and *D-mib^1^/D-mib^3^* (lane 5) third instar larvae. The D-mib protein produced in transfected S2 cells from the cDNA used in this study (lane 1) runs exactly as endogenous D-mib (lane 2). Panel C′ shows a Red Ponceau staining of the gel with the same protein samples as in panel C. (D–H) Wings from wild-type (D), *D-mib^1^* (E), *Ser^RX82^/Ser^rev6.1^* (F), *D-mib^2^/D-mib^4^* (G), and UAS-D-mib^2^/+; *D-mib^1^/D-mib^2^* flies (H). *D-mib* (E) and *Ser* (F) mutant flies showed similar wing loss phenotypes. The *D-mib* mutant phenotype could be almost fully rescued by a leaky UAS-D-mib transgene (H). (D′) and (G′) show high magnification views of (D) and (G), respectively, to show that *D-mib^2^/D-mib^4^* mutant flies (G′) exhibited ectopic sensilla (arrowheads) along vein L3. (I–N) Nota (I–K) and legs (L–N) from wild-type (I and L), *D-mib^1^* (J and M), and *Ser^RX82^/Ser^rev6.1^* (K and N) flies. *D-mib* mutant flies showed a weak neurogenic phenotype (J) that was not observed in *Ser* mutant flies (K). Ectopic sensory organs in *D-mib* mutant flies developed from ectopic sensory organ precursor cells (not shown). *D-mib* (M) and *Ser* (N) mutant legs also showed distinct growth and/or elongation defects. Arrows in (J) show ectopic macrochaetes. Arrows in (L–N) indicate the joints. Ti, tibia; t1 to t5, tarsal segments 1 to 5.

These four mutations identify the CG5841 gene as *D-mib* by the following evidence. First, lethality of homozygous *D-mib^1^* pupae is associated with the insertion of a P-element into the 5′ UTR of the *D-mib* gene. Second, genomic sequencing of the *D-mib^3^* allele revealed the presence of a stop codon at position 258 (Figure 1B). This allele is therefore predicted to produce a truncated protein devoid of the catalytic RING domain, consistent with *D-mib^3^* being a null allele. Genomic sequencing of the *D-mib^4^* allele showed that this mutation is associated with a valine-to-methionine substitution at a conserved position in the second Mib repeat (Figure 1B). Third, Western blot analysis showed that the D-mib protein was not detectable in imaginal disc and brain complex extracts prepared from homozygous *D-mib^1^* and *D-mib^1^*/*D-mib^2^* larvae (Figure 1C and C′). Fourth, the leaky, GAL4-independent expression of a UAS-D-mib transgene fully rescued the lethality of *D-mib^1^*/*D-mib^2^* flies (data not shown; see also Figure 1H). Thus, our analysis identified both complete and partial *D-mib* loss-of-function alleles.

### *D-mib* Regulates Dl Signaling

Complete loss of zygotic *D-mib* activity in homozygous *D-mib^1^* and *trans*-heterozygous *D-mib^2^*/*D-mib^3^*, *D-mib^1^*/*D-mib^3^* and *D-mib^1^*/*D-mib^2^* individuals led to late pupal lethality. Mutant pupae died as pharate adults showing ectopic macrochaetes, increased microchaete density on the dorsal thorax (Figure 1I and 1J), short legs lacking tarsal segmentation (Figure 1L and 1M), and nearly complete loss of eye and wing tissues (Figure 1D and 1E). Tissue losses were associated with a dramatic reduction in size of the eye field and of the wing pouch in mutant discs of third instar larvae (Figure 2A–2E). Hypomorphic *D-mib^2^*/*D-mib^4^* mutant flies only showed ectopic sensory organs, rough eyes, small wings, and thickened veins (Figure 1D, 1D′, 1G, and 1G′; data not shown).

**Figure 2:**
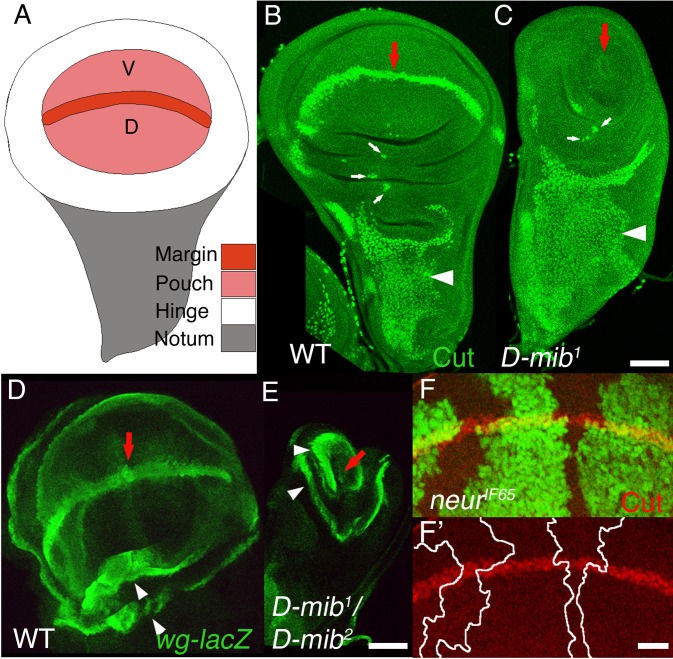
The *D-mib* and *neur* Genes Have Distinct Functions during Wing Development (A–E) Wing imaginal discs (B–E) from wild-type (B and D), *D-mib^1^* (C), and *D-mib^1^/D-mib^2^* (E) third instar larvae stained for Cut (B and C) and *wg-lacZ* (D and E). *D-mib* mutant discs showed a dramatically reduced size of the wing pouch (see diagram in [A] showing the different regions of the wing imaginal disc; V, ventral; D, dorsal), as well as a complete loss of Cut and *wg-lacZ* (red arrows in [B–E]) expression at the wing margin. Expression of *wg-lacZ* in the hinge region (arrowheads in [D] and [E]) and the accumulation of Cut in sensory cells (small arrows in [B] and [C]) and muscle precursor cells (large arrowheads in [B] and [C]) appeared to be largely unaffected). (F and F′) Expression of Cut (red) at the wing margin was not affected by the complete loss of *neur* activity in *neur^1F65^* mutant clones (indicated by the loss of the nuclear green fluorescent protein [GFP] marker, in green). Bar is 50 μm in (B–E) and 20 μm in (F and F′).

All these phenotypes may result from reduced N signaling. More specifically, the bristle and leg phenotypes are likely to result from reduced signaling by Dl (and not by Ser). Indeed, a reduction in Dl-mediated lateral inhibition can result in ectopic sensory organs and increased bristle density on the body surface. In contrast, a complete loss of Ser signaling had no effect on bristle density (Figure 1K). Likewise, loss of Dl signaling has been shown to result in short unsegmented legs, similar to the ones seen in the absence of *D-mib* activity (Figure 1M), whereas a complete loss of *Ser* activity led to the formation of elongated unsegmented legs (Figure 1N) [36,37,38]. Finally, the vein phenotype seen in *D-mib* hypomorphic flies is similar to the one seen in *Dl^ts^* mutant flies [39]. Together, these observations suggest that D-mib regulates Dl signaling in several developmental contexts. Consistent with this conclusion, we have shown that D-mib binds Dl and promotes Dl signaling and that overexpression of D-mib down-regulates the accumulation of Dl at the cell surface (E. C. Lai, F. Roegiers, X. Qin, R. Le Borgne, F. Schweisguth, et al., unpublished data).

### *D-mib* and *neur* Have Distinct Functions

We then studied in more detail the function of *D-mib* during wing development. Growth of the wing pouch depends on the activity of an organizing center located at the dorsal-ventral (D-V) boundary [40,41]. This boundary is established in first instar larvae and is defined by the *apterous* expression boundary. Apterous activates the expression of the *Ser* and *fringe* genes in dorsal cells. High levels of Ser in dorsal cells activate N in *trans* in ventral cells and suppress N activation in *cis* in dorsal cells, whereas Fringe modifies N in dorsal cells such that dorsal cells located at the D-V boundary respond to Dl. Thus, composite signaling by Ser and Dl leads to symmetric N activation in margin cells located along the D-V boundary [8,9,42,43]. N then regulates the expression of the *vestigial* and *wingless (wg)* genes that cooperate to promote growth of the wing pouch. N also regulates expression of the *cut* gene in margin cells [44]. Thus, loss of N signaling results in a reduction in size of the wing pouch accompanied by the loss of *cut* and *wg* expression along the D-V boundary.

A complete loss of Cut and Wg accumulation and *wg-lacZ* expression was observed in the central region of third instar *D-mib* mutant wing discs (data not shown). Thus, the *D-mib* wing phenotype may result from defective N inductive signaling at the D-V boundary. We conclude that the activity of the *D-mib* gene is required for the specification of the wing margin and, hence, growth of the wing pouch. Interestingly, wing margin formation and expression of Cut are not affected by the complete loss of *neur* activity (Figure 2F and 2F′) [45]. Similarly, loss of *neur* activity had no detectable effect on leg segmentation (data not shown) and vein determination [45], two processes shown here to depend on *D-mib* gene activity. We therefore conclude that *D-mib* and *neur* have distinct and complementary functions in *Drosophila*.

### D-mib Co-Localizes with Dl and Ser at the Apical Cortex

We next studied the subcellular localization of D-mib (Figure 3). Anti-D-mib antibodies were generated that specifically detected D-mib on Western blots (see Figure 1C) and on fixed tissues (Figure 3F–F″). Using these antibodies, we found that D-mib was detected in all imaginal disc cells (Figure 3A and 3B). We then examined D-mib subcellular distribution in epithelial cells located along the edge of the wing discs because cross-sectional imaging affords better resolution along the apical-basal axis. D-mib co-localized with Ser, Dl, and N at the apical cortex (Figure 3B–3D′′′). Dl and Ser were also detected in large intracellular vesicles that probably correspond to multivesicular bodies in that they also stained for hepatocyte growth factor-regulated tyrosine kinase substrate [46] (Figure 3B–3C′′′; data not shown). The intracellular dots seen with the anti-D-mib antibodies were distinct from the Dl- and Ser-positive dots and appeared to result from background staining (data not shown). The reduced cytoplasmic staining seen in *D-mib* mutant cells (Figure 3F–3F′′) suggests that D-mib is also present in the cytoplasm. A similar localization at the apical cortex and in the cytoplasm was seen for a functional yellow fluorescent protein (YFP)::D-mib fusion protein (see Figure 6 below). These localization data suggest that D-mib may act at the apical cortex to regulate the activity of Dl and/or Ser.

**Figure 3:**
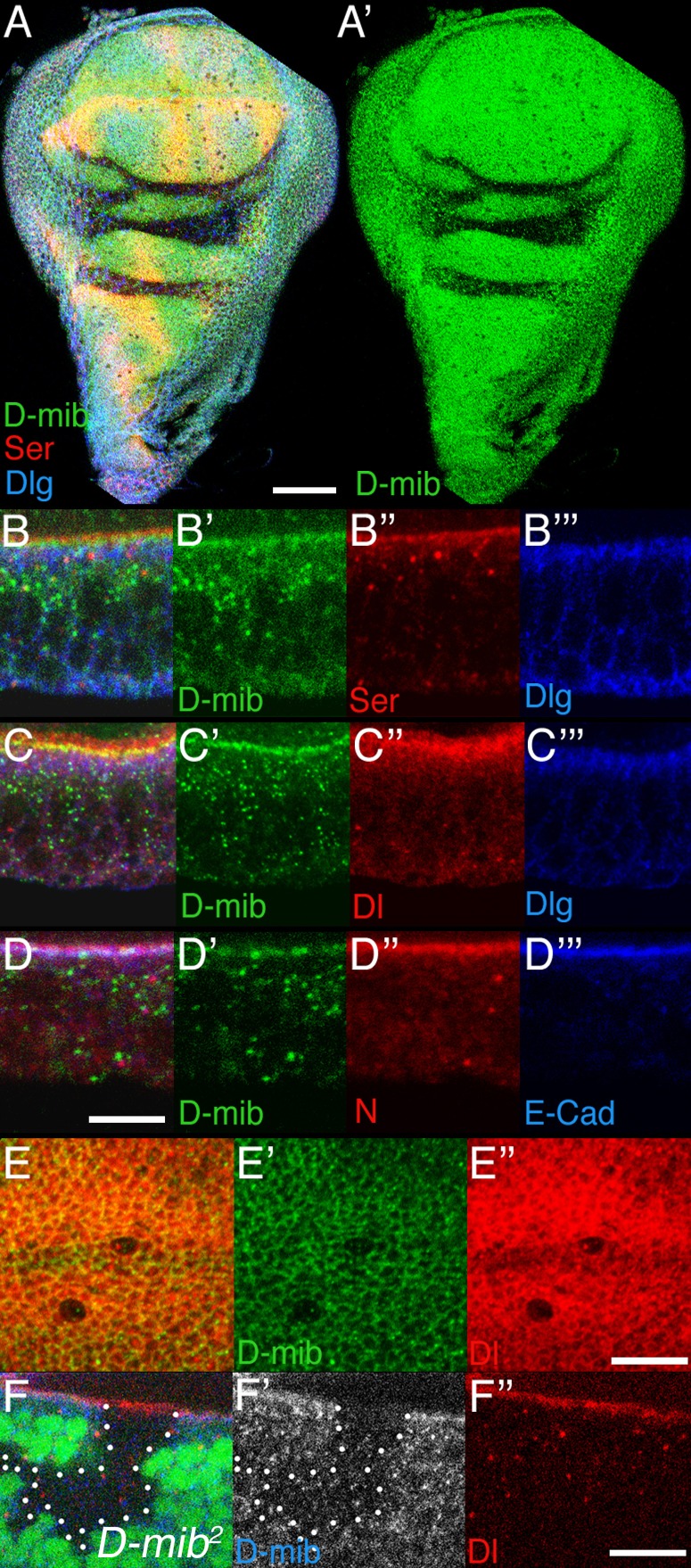
D-mib Co-Localizes with Dl and Ser at the Apical Cell Cortex (A and A′) D-mib (green) is detected in all cells of the wing imaginal disc. In (A), Ser is in red and Discs-large (Dlg) is in blue. (B–D′′′) D-mib (green in B, B′, C, C′, D, and D′) co-localized with Ser (red in [B and B′′]), Dl (red in [C and C′′]), N (red in [D and D′′]), and E-Cadherin (E-Cad; blue in [D and D′′′]) and was found apical to Discs-large (Dlg; blue in [B, B′′′, C, and C′′′]) in notum cells located at the edges of the wing discs. (E–E′′) D-mib (green in [E and E′]) co-localized with Dl (red in [E and E′′]) at the apical cortex of wing pouch cells. (F–F′′) D-mib staining at the apical cortex (blue in [F and F′]) was not detected in *D-mib^2^* mutant clone (marked by loss of nuclear GFP staining; green in [F]). Loss of *D-mib* activity has no detectable effect on the apical accumulation of Dl (red in [F and F′′]). Bar is 50 μm for (A and A′) and 10 μm for (B–F′′).

**Figure 6:**
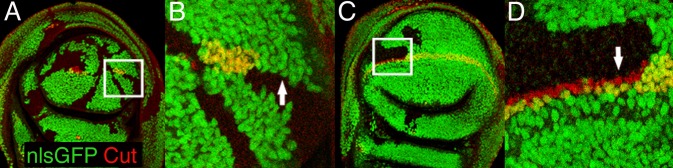
*D-mib* Is Required in Dorsal Cells for Margin Expression of Cut Large dorsal clones of *D-mib^2^* mutant cells (marked by the loss of nuclear GFP, in green) resulted in a complete loss of Cut (red) expression (A and B). This indicates that D-mib is required for Ser signaling by dorsal cells. In contrast, ventral clones did not prevent the expression of Cut (C and D), implying that D-mib is not strictly required for Dl signaling. Note that mutant ventral cells abutting wild-type dorsal cells expressed Cut (arrow in [D]), indicating that *D-mib* is not required for N signal transduction. Low-magnification views of the wing portion of the discs are shown in (A) and (C). (B) and (D) show high-magnification views of the areas boxed in (A) and (C), respectively.

### *D-mib* Regulates the Cell-Surface Level of Ser

We next examined the potential role of D-mib in regulating Dl and Ser distribution in wing imaginal discs. We focused our analysis on the notum region since *D-mib* mutant discs have no wing pouch (Figure 4). Dl and Ser co-localized both at the apical cortex and in large intracellular vesicles in wild-type cells (Figure 4A–4C′). The complete loss of *D-mib* activity in *D-mib^1^* mutant discs did not detectably change the subcellular localization of Dl (Figure 4C, 4C′, 4F, and 4F′). In contrast, the accumulation of Ser at the apical cortex was strongly increased (Figure 4E) and Ser accumulation in Dl-positive vesicles was dramatically reduced (Figure 4E′) in *D-mib^1^* mutant discs. Similar results were also obtained in *D-mib^2^* mutant clones, which showed strongly elevated levels of cortical Ser (Figure 4H) whereas the amount of Dl at the apical cortex was not detectably modified (see Figures 3F–3F′′ and 4J). Of note, loss of *D-mib^2^* activity in clones did not block the accumulation of Ser into intracellular dots (Figure 4H′). Thus, trafficking of Ser towards this intracellular compartment is, at least in part, *D-mib*-independent. We therefore conclude that the *D-mib* gene is required to regulate the level of Ser at the apical cortex of wing disc cells.

**Figure 4:**
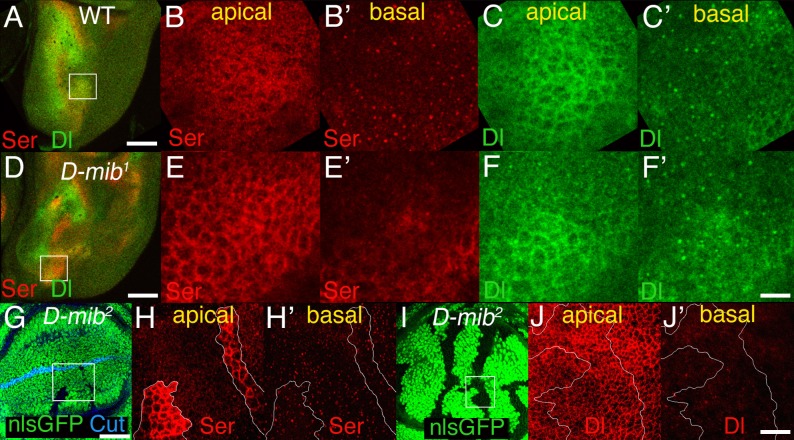
D-mib Is Required to Down-Regulate Ser at the Apical Cortex (A–F′) Distribution of Dl (green) and Ser (red) in the notum region of wild-type (A–C′) and *D-mib^1^* mutant (D–F′) wing imaginal discs. The boxed areas in (A) and (D) are shown at higher magnification in (B–F′). The specific loss of Ser accumulation into intracellular vesicles (compare [E′] with [B′]) correlated with the elevated levels of Ser seen at the apical cortex of *D-mib* mutant cells (compare [E] with [B]). (G–J′) Ser (red in [H and H′]) accumulated at the apical cortex (H) as well as in intracellular dots (H′) in *D-mib^2^* mutant cells (marked by the loss of nuclear GFP; green in [G]). Cut is shown in blue (G). The distribution of Dl (red in [J and J′]) was not affected by the loss of *D-mib* activity. Low-magnification views of the wing portion of the discs are shown in (G) and (I). (H and H′) and (J and J′) show high magnification views of the areas boxed in (G) and (I), respectively. Clone boundaries are outlined in (H and H′) and (J and J′). Bar is 40 μm for (A, D, G), 5 μm for (B–C′ and E–F′), and 10 μm for (H–J′).

### *D-mib* Is Required for Ser Endocytosis

To test whether this specific increase in the level of Ser at the apical cortex resulted from reduced Ser endocytosis in *D-mib* mutant cells, we followed the endocytosis of Ser in living imaginal discs using an antibody uptake assay. Briefly, dissected wing discs were cultured for 15 min in the presence of antibodies that recognize the extracellular part of Ser or Dl, then washed, cultured for another 45 min in medium without antibodies, and then fixed. The uptake of anti-Ser and anti-Dl antibodies was then assessed using secondary antibodies. The results are shown in Figure 5. Using this assay, we found that anti-Ser-and anti-Dl antibodies were internalized in wild-type epithelial cells (Figure 5A–5C′′). The complete loss of *D-mib* activity in *D-mib^1^* wing discs did not significantly change the internalization of anti-Dl antibodies (Figure 5D′′, 5E′′, and 5F′′), indicating that *D-mib* is not required for Dl endocytosis in this tissue. However, the loss of *D-mib* activity strongly inhibited the endocytosis of anti-Ser antibodies (Figure 5E′). Moreover, high levels of anti-Ser antibodies were seen at the apical surface (Figure 5D′ and 5F′), confirming that *D-mib* mutant cells accumulate high levels of Ser at their surface. We therefore conclude that *D-mib* is specifically required for the endocytosis of Ser in wing discs.

**Figure 5:**
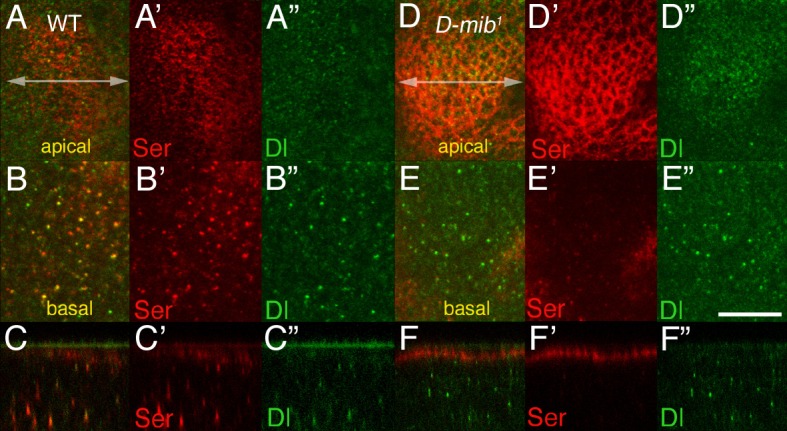
D-mib Is Required for Ser Endocytosis Localization of the anti-Ser (red) and anti-Dl (green) antibodies that have been internalized by wild-type (A–C′′) and *D-mib^1^* mutant (D–F′′) cells in the notum region of wing discs. (A–A′′) and (D–D′′) show apical sections and (B–B′′) and (E–E′′) show basal sections. (C–C′′) and (F–F′′) show confocal z-sections. The z-section axes are shown with a double-headed arrow in (A) and (D). Internalized anti-Ser and anti-Dl antibodies co-localized in wild-type cells. In contrast, high levels of anti-Ser antibodies were detected at the cell surface of *D-mib* mutant epithelial cells whereas anti-Dl antibodies were efficiently internalized. Bar is 10 μm for all panels.

Ubiquitin-mediated endocytosis is thought to depend on monoubiquitination. Thus, by analogy with the function of Mib in D. rerio [18,28], we suggest that D-mib may directly monoubiquitinate Ser. Consistent with this hypothesis, we show in a companion paper that D-mib binds Ser (E. C. Lai, F. Roegiers, X. Qin, R. Le Borgne, F. Schweisguth, et al., unpublished data). Moreover, a mutation in the C-terminal catalytic RING domain of D-mib abolished its ability to internalize Ser in transfected S2 cells (R. L. B. and F. S., unpublished data) implying that the E3 ubiquitin ligase activity of D-mib is required for Ser internalization. Biochemical analysis of the ubiquitination events regulated by D-mib will be needed to further define the mechanism by which D-mib regulates the endocytosis of Ser in vivo.

### *D-mib* Regulates Ser Signaling

The regulation of Ser endocytosis by D-mib suggests that D-mib may regulate Ser signaling. Ser expression is restricted to dorsal cells in second instar wing imaginal discs [7,10,44,47,48]. Ser in dorsal cells signals across the D-V boundary to activate N in ventral cells [8,9]. If *D-mib* is required for Ser signaling during wing development, then loss of *D-mib* activity in dorsal cells should affect the specification of the wing margin in a non-autonomous manner. Loss of *D-mib* activity in large dorsal clones of *D-mib^2^* mutant cells resulted in a loss of Cut expression at the D-V interface (Figure 6A and 6B). The lack of Cut expression in wild-type ventral cells abutting the D-V boundary indicates that *D-mib* is required for Ser signaling by dorsal cells and acts in a non-autonomous manner to activate N in ventral cells. Conversely, loss of *D-mib* activity in large ventral clones (Figure 6C and 6D) did not disrupt margin specification, indicating that *D-mib* is not strictly required for Dl signaling by ventral cells. However, a narrowing of the Cut-positive margin was observed (Figure 6D), suggesting that D-mib contributes to regulating the level of Dl signaling. Of note, ventral *D-mib* mutant cells expressed Cut, implying that D-mib is not required for N signal transduction.

We next tested whether expression of *D-mib* in dorsal cells is sufficient to rescue the *D-mib* wing phenotype. D-mib was expressed in dorsal cells of *D-mib^2^*/*D-mib^3^* mutant discs using Ser-GAL4. Similarly to the expression of the *Ser* gene, Ser-GAL4 expression is restricted to dorsal cells in second/early third instar larvae and is weakly expressed in ventral cells in mid/late third instar larvae, i.e., after margin cell specification [49,50]. Expression of D-mib in dorsal cells was sufficient to rescue growth of the wing pouch and of the expression of Cut in margin cells in *D-mib* mutant discs (Figure 7A). This result confirmed that D-mib regulates Ser signaling by dorsal cells.

**Figure 7:**
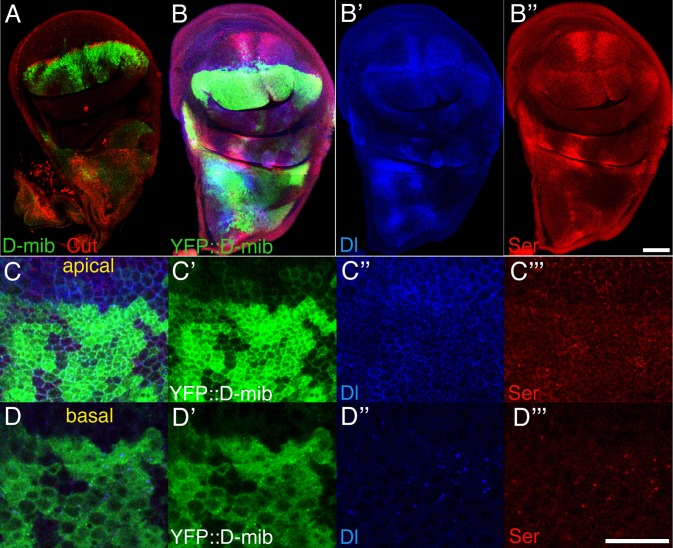
Expression of D-mib in Dorsal Cells Is Sufficient to Rescue the *D-mib* Mutant Phenotype (A) Expression of D-mib (green) in dorsal cells, using Ser-GAL4, rescued the growth of the wing pouch and margin Cut (red) expression in *D-mib^2^*/*D-mib^3^* mutant discs. (B–D′′′) Ser-GAL4-driven expression of YFP::D-mib (green) rescued the *D-mib^2^*/*D-mib^3^* phenotype and strongly reduced the level of Dl (blue in [B, B′, C, C′′, D, and D′′]) and Ser (red in [B, B′′, C, C′′′, D, and D′′′]) in dorsal cells. (C–D′′′) are high-magnification views (apical [C–C′′′] and basal [D–D′′′]) of the disc shown in (B–B′′). YFP::D-mib co-localized with Dl and Ser at the apical cortex in cells expressing only low levels of YFP::D-mib. Bar is 50 μm for (A–B′′) and 10 μm for (C–D′′′).

A similar rescue was observed with a YFP::D-mib protein (Figure 7B–7B″), indicating that YFP::D-mib is functional. YFP::D-mib localized at the apical cortex and in the cytoplasm (Figure 7C–7 D′′′), as seen for endogenous D-mib (see Figure 3). YFP::D-mib co-localized with Dl and Ser at the apical cortex of cells expressing low levels of YFP::D-mib. However, cells expressing high levels of YFP::D-mib showed a strong reduction in the level of both Dl and Ser at the cortex (Figure 7C–7C′′′), further indicating that D-mib down-regulates the levels of both Ser and Dl at the apical cortex (E. C. Lai, F. Roegiers, X. Qin, R. Le Borgne, F. Schweisguth, et al., unpublished data).

### *D-mib* Acts Downstream of Ser and Upstream of Activated N

The functional assay was then used to genetically position the requirement for the *D-mib* gene activity relative to Ser and N (Figure 8). Expression of an activated version of N, Ncdc10 [51], led to the activation of Cut and promoted growth in dorsal cells of *D-mib^2^*/*D-mib^3^* mutant discs (Figure 8C). This indicates that D-mib acts at a step upstream of N activation. By contrast, elevated levels of Ser expression failed to restore Cut expression and growth of the wing pouch in *D-mib^2^*/*D-mib^3^* mutant larvae (Figure 8B). This confirms that Ser signaling requires the activity of the *D-mib* gene, i.e., that D-mib acts downstream of Ser.

**Figure 8:**
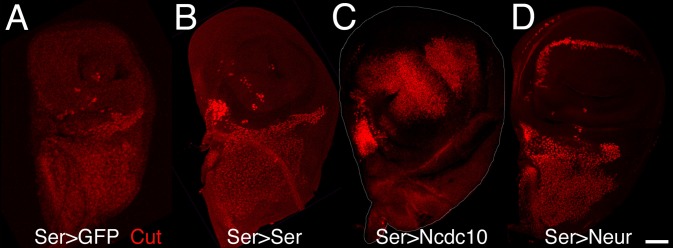
Expression of Neur in Dorsal Cells Is Sufficient to Rescue the *D-mib* Mutant Phenotype *D-mib^2^*/*D-mib^3^* mutant discs expressing GFP (A) (GFP staining not shown), Ser (B), Ncdc10 (C), or Neur (D) under the control of Ser-GAL4 were stained for Cut (red). Expression of Ser in dorsal cells did not rescue the *D-mib^2^*/*D-mib^3^* wing pouch mutant phenotype (compare [B] with [A]), consistent with *D-mib* being required for Ser signaling. By contrast, expression of Ncdc10, an activated version of N, led to the deregulated growth of the dorsal compartment and the expression of Cut in most dorsal cells (C), indicating that activated N acts downstream of *D-mib*. Expression of Neur in dorsal cells was sufficient to compensate for the loss of *D-mib* activity (D). Bar is 40 μm for all panels.

### *neur* and *D-mib* Functions Partially Overlap

The different requirements for *neur* and *D-mib* gene activity may suggest that Neur and D-mib have distinct molecular activities. Alternatively, this difference may reflect a difference in gene expression. Consistent with the latter hypothesis, the *neur* gene is not expressed in wing pouch and wing margin cells, where it is not required, and appears to be expressed only in sensory cells [52], where it is required. By contrast, D-mib appears to be uniformly expressed in imaginal discs. To test this hypothesis, we examined whether the forced ubiquitous expression of the *neur* gene can suppress the *D-mib* loss-of-function phenotype. Expression of Neur, using *actin*-GAL4, restored growth of the wing pouch and formation of the wing margin (data not shown). Moreover, expression of Neur in dorsal cells, using Ser-GAL4, was sufficient to rescue growth of the wing pouch as well as the expression of Cut in margin cells in *D-mib* mutant discs (Figure 8D). We conclude that ectopic expression of Neur compensates for the loss of *D-mib* activity.

In a converse experiment, we found that the *neur*-driven expression of *D-mib,* using *neur^PGAL4^,* did not rescue the cuticular neurogenic phenotype of *neur^PGAL4^*/*neur^1F65^* embryos. Three UAS-D-mib transgenic lines were tested, and none showed detectable rescue whereas the two UAS-neur lines used as positive controls either fully or partially rescued the cuticular neurogenic phenotype of *neur^PGAL4^*/*neur^1F65^* embryos (data not shown; UAS-D-mib*/+*, *neur^PGAL4^*/+ embryos developed normally). This indicates that a key function of Neur in the embryo cannot be provided by D-mib. We therefore suggest that Neur and D-mib functions overlap but are not strictly identical.

## Discussion

Many recent studies have revealed that endocytosis plays multiple roles in the regulation of N signaling (reviewed in [2]; see also [53,54]). Here, we show that the conserved E3 ubiquitin ligases Neur and D-mib have similar molecular activities in the regulation of Dl and Ser endocytosis but distinct developmental functions in *Drosophila*.

Our analysis first establishes that D-mib regulates Ser signaling during wing development. First, clonal analysis revealed that the activity of the *D-mib* gene is specifically required in dorsal cells for the expression of Cut at the wing margin. Second, expression of *D-mib* in the dorsal Ser-signaling cells was sufficient to rescue the *D-mib* mutant wing phenotype. Third, results from an in vivo antibody uptake assay indicated that the endocytosis of Ser (but not of Dl) was strongly inhibited in *D-mib* mutant cells. This inhibition correlated with the strong accumulation of Ser (but not Dl) at the apical cortex of *D-mib* mutant cells. Thus, an essential function of *D-mib* in the wing is to regulate the endocytosis of Ser in dorsal cells to non-autonomously promote the activation of N along the D-V boundary. By analogy, the defective growth of the eye tissue may similarly result from the lack of Ser signaling and of N activation along the D-V boundary [55]. Because D-mib co-localizes with Ser at the apical cortex of wing disc cells, acts in a RING-finger-dependent manner to regulate Ser endocytosis in S2 cells (R. L. B. and F. S., unpublished results), and physically associates with Ser in co-immunoprecipitation experiments (E. C. Lai, F. Roegiers, X. Qin, R. Le Borgne, F. Schweisguth, et al., unpublished data), D-mib may ubiquitinate Ser and directly regulate its endocytosis.

Our analysis further suggests that endocytosis of Ser is required for Ser signaling. This conclusion is consistent with observations made earlier showing that secreted versions of Ser cannot activate N but instead antagonize Ser signaling [56,57]. Thus, endocytosis of both N ligands appears to be strictly required for N activation in *Drosophila*. Different models have been proposed to explain how endocytosis of the ligand, which removes the ligand from the cell surface, results in N receptor activation (discussed in [17,20,21,30]). Interestingly, the strong requirement for Dl and Ser endocytosis seen in *Drosophila* is not conserved in *Caenorhabditis elegans,* in which secreted ligands have been shown to be functional [58,59]. Noticeably, there is no C. elegans Mib homolog, and the function of *C. elegans neur* (F10D7.5) is not known. We speculate that endocytosis of the ligands may have evolved as a means to ensure tight spatial regulation of the activation of N.

Our analysis also establishes that the activity of the *D-mib* gene is required for a subset of N signaling events that are distinct from those that require the activity of the *neur* gene. We have shown that the *D-mib* gene regulates wing margin formation, leg segmentation, and vein formation, whereas none of these three processes depend on *neur* gene activity ([45,60]; this study). Conversely, the activity of the *neur* gene is essential for binary cell-fate decisions in the bristle lineage [22] that do not require the activity of the *D-mib* gene (no bristle defects were seen in *D-mib* mutant flies). The activity of the *neur* gene is also required for lateral inhibition during neurogenesis in embryos and pupae [4,45,61]. This process is largely independent of *D-mib* gene activity since the complete loss of *D-mib* function only resulted in a mild neurogenic phenotype in the notum. These data thus indicate that the *neur* and *D-mib* genes have largely distinct and complementary functions in *Drosophila*. Whether a similar functional relationship between Neur and D-mib exists in vertebrates awaits the study of the *D. rerio neur* genes and/or of the murine *Mib* and *Neur* genes.

The functional differences observed between *D-mib* and *neur* cannot be simply explained by obvious differences in molecular activity and/or substrate specificity. First, both Neur and D-mib physically interact with Dl ([20]; E. C. Lai, F. Roegiers, X. Qin, R. Le Borgne, F. Schweisguth, et al., unpublished data) and promote the down-regulation of Dl from the apical membrane when overexpressed (E. C. Lai, F. Roegiers, X. Qin, R. Le Borgne, F. Schweisguth, et al., unpublished data). Furthermore, Dl signaling appears to require the activity of either Neur or D-mib, depending on the developmental contexts. We have shown here that specific aspects of the *D-mib* phenotype in legs and in the notum cannot simply result from loss of Ser signaling and are consistent with reduced Dl signaling, suggesting that D-mib regulates Dl signaling. Consistent with this interpretation, overexpression studies indicate that D-mib up-regulates the signaling activity of Dl, whereas a dominant-negative form of D-mib inhibits it (E. C. Lai, F. Roegiers, X. Qin, R. Le Borgne, F. Schweisguth, et al., unpublished data). We note, however, that no clear defects in Dl subcellular localization and/or trafficking were observed in *D-mib* mutant cells. It is conceivable that the contribution of D-mib to the endocytosis of Dl is masked by the activity of *D-mib*-independent processes that may, or may not, be linked to Dl signaling. We have also shown that, reciprocally, Neur and D-mib may similarly regulate Ser. Neur and D-mib were shown to similarly promote down-regulation of Ser from the cell surface when overexpressed (E. C. Lai, F. Roegiers, X. Qin, R. Le Borgne, F. Schweisguth, et al., unpublished data). Moreover, D-mib binds Ser (E. C. Lai, F. Roegiers, X. Qin, R. Le Borgne, F. Schweisguth, et al., unpublished data) and regulates Ser signaling (this study). Whether endogenous Neur binds and activates Ser remains to be tested. However, the ability of Neur to rescue the *D-mib* mutant wing phenotype when expressed in dorsal cells strongly indicates that Neur can promote Ser signaling. Together, these data indicate that Neur and D-mib have similar molecular activities.

D-mib and Neur may have identical molecular activities but distinct expression patterns, hence distinct functions at the level of the organism. Consistent with this possibility, D-mib is uniformly distributed in imaginal discs, whereas Neur is specifically detected in sensory cells [52]. Importantly, the rescue of the *D-mib* mutant phenotype by ectopic expression of Neur strongly supports this interpretation. This result further suggests that Neur can regulate Ser signaling. Consistent with this idea, overexpression of Neur in imaginal discs resulted in a strong reduction of Ser accumulation at the apical cortex (data not shown). Thus, despite their obvious structural differences, Neur and D-mib appear to act similarly to promote the endocytosis of Dl and Ser. Nevertheless, our observation that D-mib could not compensate for the loss of *neur* activity in the embryo indicates that D-mib and Neur have overlapping rather than identical molecular activities.

In conclusion, Neur and D-mib appear to have similar molecular activities in the regulation of Dl and Ser endocytosis but distinct developmental functions in *Drosophila*. The conservation from *Drosophila* to mammals of these two structurally distinct but functionally similar E3 ubiquitin ligases is likely to reflect a combination of evolutionary advantages associated with: (i) specialized expression pattern, as evidenced by the cell-specific expression of the *neur* gene in sensory organ precursor cells [52]; (ii) specialized function, as suggested by the role of murine MIB in TNFα signaling [32]; (iii) regulation of protein stability, localization, and/or activity. For instance, Neur, but not D-mib, localizes asymmetrically during asymmetric sensory organ precursor cell divisions [22].

## Materials and Methods

### 

#### Flies

The *D-mib^1^* mutation corresponds to the EY97600 P-element insertion generated by the Gene Disruption Project (http://flypush.imgen.bcm.tmc.edu/pscreen/). The *D-mib^2^* allele was selected as *w^−^ D-mib* mutant derivative by imprecise excision of the EY97600 P-element. The precise breakpoints of the *D-mib^2^* deletion were determined by sequencing a PCR fragment amplified from genomic DNA prepared from *D-mib^2^* homozygous larvae. The *l(3)72Cda^J12^* and *l(3)72Cda^I5^* alleles originally isolated by [35] failed to complement the *D-mib^1^* and *D-mib^2^* mutations and were renamed *D-mib^3^* and *D-mib^4^*. The *D-mib^1^, D-mib^2^,* and *D-mib^3^* alleles appear to be genetically null alleles since the phenotypes of *D-mib^1^*/*D-mib^1^* and *D-mib^1^*/*D-mib^3^* mutant pupae are indistinguishable from the ones seen in *D-mib^1^*/*D-mib^2^* and *D-mib^2^*/*D-mib^3^* pupae. Sequence analysis of the *D-mib^3^* and *D-mib^4^* alleles was carried on PCR products prepared from genomic DNA prepared from *D-mib^3^/D-mib^2^* and *D-mib^4^*/*D-mib^2^* mutant pupae. Genomic DNA from *l(3)72Cda/D-mib^2^* mutant pupae was used as control for polymorphism.

*D-mib^2^* mutant clones were generated in *y w* hs-flp;FRT2A *D-mib^2^*/FRT2A *M(3)^i55^* ubi-nlsGFP larvae. *neur^1F65^* mutant clones were generated as previously described [22].

UAS-D-mib and UAS-YFP::D-mib lines were generated via standard P-element transformation. These constructs were derived from the SD05267 cDNA obtained from ResGen (Invitrogen, Carlsbad, California, United States). Cloning details for these constructs are available upon request. UAS-Dl (gift from M. Muskavith), UAS-Ser (gift from R. Fleming), UAS-Neur (gift from C. Delidakis), UAS-Ncdc10 (gift of T. Klein), Ser-GAL4 lines, and *Ser* mutant alleles are described in FlyBase (http://flybase.bio.indiana.edu/).

#### Antibodies

Dissected imaginal discs were fixed in 4% paraformaldehyde (15 min) and incubated with antibodies at room temperature in PBS 1X with 0.1% TritonX-100. Rabbit polyclonal anti-D-mib antibodies were raised against the CYNERKTDDSELPGN peptide (CovalAb, Lyon, France). Immunopurified anti-D-mib antibodies (rabbit 541) were used (immunofluorescence, 1:100; Western blot, 1:1,000). Other primary antibodies were mouse anti-Cut (2B10; Developmental Studies Hybridoma Bank [DSHB, Iowa City, Iowa, United States]; 1:500); rat anti-DE-Cadherin (gift from T. Uemura; 1:50); guinea pig anti-Discs-large (gift from P. Bryant; 1:3,000); anti-β-galactosidase (Cappel [MP Biomedicals, Irvine, California, United States]; 1:1,000); mouse anti-DeltaECD (C594.9B; DSHB; 1:1,000); mouse anti-NotchECD (C548.2H; DSHB; 1:1,000); rat anti-Ser (gift from K. Irvine; 1:2,000); rat anti-Ser (gift from S. Cohen; 1:200); rabbit anti-Ser (gift from E. Knust; 1:10); and guinea pig anti-Senseless (gift from H. Bellen; 1:3,000). Cy2-, Cy3-, and Cy5-coupled secondary antibodies were from Jackson Laboratory (Bar Harbor, Maine, United States). Alexa488-coupled secondary antibodies and phalloidin were from Molecular Probes (Eugene, Oregon, United States). Images were acquired on a Leica (Wetzlar, Germany) SP2 microscope and assembled using Adobe Photoshop (Adobe Systems, San Jose, California, United States).

#### Endocytosis assay

Third instar larvae wing discs were dissected in Schneider's *Drosophila* medium (Gibco BRL, San Diego, California, United States) containing 10% fetal calf serum (Gibco BRL). Wing discs were cut between the wing pouch and the thorax to facilitate antibody diffusion. Wing discs were cultured for 15 min with mouse anti-Dl (C594–9B at 1:100) and rat anti-Ser antibody (1:500; from K. Irvine). Following three medium changes and a 45-min chase period, wing discs were fixed and incubated with secondary antibodies.

## Supporting Information

### Accession Numbers

The FlyBase accession numbers for the gene products discussed in this paper are Dl (FBgn0000463), N (FBgn0004647), Neur (FBgn0002932), P-element inserted into the 5′ untranslated region of the *D-mib* gene (FBgn0036558), and Ser (FBgn0004197). The NCBI Entrez Protein (http://www.ncbi.nlm.nih.gov/entrez/) accession number for D. rerio Mib is NP_779353.

## Abbreviations

Dl: Delta
*D-mib*: *Drosophila mind bomb*
DSHB: Developmental Studies Hybridoma Bank
D-V: dorsal-ventral
GFP: green fluorescent protein
Mib: Mind bomb
N: Notch
Neur: Neuralized
RING: really interesting new gene
Ser: Serrate
wg: wingless
YFP: yellow fluorescent protein
